# Photobiomodulation: A Systematic Review of the Oncologic Safety of Low-Level Light Therapy for Aesthetic Skin Rejuvenation

**DOI:** 10.1093/asj/sjad018

**Published:** 2023-02-01

**Authors:** Graeme Ewan Glass

## Abstract

Photobiomodulation (PBM) therapy is an increasingly popular modality for aesthetic skin rejuvenation. PBM induces genomic, proteomic, and metabolomic processes within target cells, but such manipulation of cell behavior has led to concerns about oncologic safety. This article presents a summary of the clinical and preclinical evidence for the oncologic safety of PBM for aesthetic skin rejuvenation. A focused systematic review was performed, in which safety data from clinical trials of PBM for skin rejuvenation was supplemented by analyses of in vitro data obtained from cells derived from human skin and human neoplastic cells and in vivo data of tumors of the skin, oral cavity, and breast. Within established parameters, red and near infrared light mainly enhances proliferation of healthy cells without a clear pattern of influence on cell viability. The same light parameters mainly reduce neoplastic cell proliferation and viability or else make no difference. Invasiveness potential (appraised by cell migration assays and/or differential gene expression) is equivocal. PBM does not induce dysplastic change in healthy cells. In vivo tumor models yield varied results with no clear pattern emerging. There are no relevant clinical trial data linking PBM with any significant adverse events, including the finding of a new or recurrent malignancy.

Current clinical and preclinical evidence suggests that PBM is oncologically safe for skin rejuvenation, and there is no evidence to support the proposition that it should be avoided by patients who have previously undergone treatment for cancer.

**Level of Evidence: 4:**

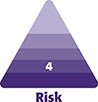

Photobiomodulation (PBM), synonymous with low-level light (laser) therapy (LLLT) has gained traction as a noninvasive therapy for skin rejuvenation, including treatment of facial rhytids, dyschromias, and acne vulgaris; wound healing including scar management; body contouring (either alone or as a means of enhancing the removal of fat during liposculpture); and androgenic alopecia.^1^ The therapeutic potential of PBM is becoming generally accepted as clinical trial data continues to provide evidence of efficacy and safety. However, controversies remain.^2-4^ One of the most pertinent discussion points is the issue of oncologic safety, with theoretical concerns expressed on account of the upregulation of cellular metabolic activity on exposure to red and near infrared laser light.^5-8^

The reason for taking this question seriously lies in the complex physiological responses to low-level laser light. Red and near infrared light energy is absorbed by intramitochondrial chromophores including cytochrome c oxidase (CcO) and perhaps one or more flavoenzymes. The absorption of light by CcO (an important enzyme in the electron transport chain) facilitates electron transport and drives adenosine triphosphate (ATP) synthesis. ATP, in turn, drives cell metabolomics.^9^ Moreover, in a localized environment of oxidative stress, red and near infrared light dissociates nitric oxide (NO), competitively bound to CcO at the expense of oxygen, restoring functionality to the electron transport chain and releasing NO to induce localized vasodilation, thereby improving localized oxygen delivery and the migration of cells of innate immunity. Simultaneously the absorption of light by flavoenzymes such as flavin dehydrogenase alters the reduction/oxidation (redox) state of the intracellular microenvironment. Not only does the balance of reactive oxygen species influence mitochondrial ATP production as detailed above, it alters the transcriptome to favor the expression of genes implicated in regeneration and repair and, thereby, the recruitment of cells of innate and adaptive immunity implicated in the coordination of tissue repair.^10-13^ A more detailed description of the role of PBM on cell signaling, immune modulation, and regulation of microcirculation is provided elsewhere.^14,15^

In a sense we have now come full circle, because the first experiments with low-energy red laser light were actually performed for the purpose of establishing oncologic safety over 50 years go; experiments that yielded incidental evidence of accelerated hair regrowth and enhanced wound healing but, it should be noted, no evidence of neoplastic change.^16,17^ With a large body of clinical and experimental evidence now behind us and the popularity of PBM booming, it is time to reexamine the evidence to establish whether this therapy is safe for our aesthetic patients, including those who have previously undergone treatment for cancer or those with an undiagnosed dysplasia or neoplasia, and to enable us to address the queries and concerns of our well-informed and curious patients.

## METHODS

### Search Strategy

A literature review was conducted to identify articles pertaining to the oncologic safety of PBM when utilized for aesthetic skin rejuvenation, including scar management. The study was performed by GG and repeated by AH and JB (see acknowledgments). Thus, 3 independent searches were performed. Discrepancies were handled by the sole author (GG), who made the final decisions on source inclusion. The search was conducted with Google Scholar (1997 to May 2022; Google, Mountain View, CA); PubMed (1997 to October 2022; US National Library of Medicine, Bethesda, MD); OVID Medline (January 1997 to October 2022; Wolters Kluwer, Alphen van den Rijn, the Netherlands); and the Cochrane database of systematic reviews and the Cochrane controlled trials register (searched July 18, 2022; Wiley, Hoboken, NJ). A 25-year range was chosen because it was felt that this best encapsulated the contemporary literature on the subject. Both preclinical (experimental) in vitro and in vivo studies and clinical trials were included. The search was performed in accordance with Preferred Reporting Items for Systematic Reviews and Meta-Analyses (PRISMA).^18^ The search strategy is summarized in Supplemental Table 1 and the PRISMA checklist is provided in Supplemental Table 2.

### Inclusion Criteria

All evidence was sought for the oncologic safety of PBM when utilized for skin rejuvenation. In vitro studies were included if they employed human primary cells, stem cells, or tumor cell lines derived from skin, breast, fat, salivary or oral mucosa, endothelium, or hair follicles. These included mesenchymal stem cells, adipocytes, fibroblasts, keratinocytes, melanocytes, Langerhans cells, endothelial cells, ductal epithelial cells, and lobular epithelial cells and the associated precursor cells.

With in vivo studies, investigators address the shortcomings of in vitro study design by examining tumor cell response in the context of tissue architecture and host immune response. By their nature, most in vivo studies use animal models and cell lines. However, when the cell line and in vivo model afforded the opportunity of a direct comparison with a neoplastic process of clinical relevance to the use of PBM, the inclusion of such studies was thought to be relevant. In vivo studies of relevance to our investigation included oral squamous cell carcinoma (and other tumors of the head and neck); melanoma; and breast (adeno)carcinoma. The fact that this was the only section containing animal data meant that it could be evaluated on its own merits without clouding the overall picture.

Clinical trials were included if the data they contained related to the oncologic safety of PBM or could be extrapolated to draw inferences about oncologic safety when employed for skin rejuvenation. Only PBM applied with light of wavelengths at the red (625-700 nm) and infrared (700-1100 nm) end of the spectrum were included, because this represented the spectrum of light utilized in PBM for skin rejuvenation commercially.

### Exclusion Criteria

In vitro studies utilizing nonhuman cells and nonhuman (veterinarian) clinical trials were excluded. Similarly, in vitro and in vivo studies using primary cells or tumor cell lines with no direct or indirect correlation to skin rejuvenation (eg, diabetic fibroblasts and keratinocytes, bone marrow–derived stem cells, endothelial cells) and nonrelevant clinical studies (eg, PBM for radiation mucositis) were excluded. Studies that did correlate with skin rejuvenation but reported only on the efficacy of PBM and not on oncologic safety were also excluded because they have been considered elsewhere.^1^ Only experimental data were considered; therefore reviews were excluded from the analyses. Thermal light exposures, including high-energy laser, intermittent pulsed light, and photodynamic therapy, as well as PBM with light of wavelengths other than those in the red/near infrared end of the spectrum were also excluded. Low quality clinical trials, letters, or short reports that relied exclusively on subjective data were not considered.

### Data Extraction

In accordance with the recommendations of Jenkins and Carroll (2011) on the minimum reporting standards for PBM, the following irradiation parameters were noted when available: source of light, wavelength, power, beam area, duration of exposure (therefore, by calculation, fluence), and continuous or pulsed wave, as well as the characteristics of the study including participants, therapeutic protocol, objective outcome measures, and findings.^19^ The nature of the clinical safety data or the experimental data on which safety conclusions could be drawn was noted.

### Data Analysis

Following extraction of the raw data, analysis revealed a large number of variables with respect to both light and experimental model. These included light source, wavelength, power, fluence (energy per unit area), and exposure protocol; and model cell type and source, species, culture conditions including the presence of additional experimental variables, experimental protocol, and methods of evaluation of effect.

For this reason, it was felt that an attempt to perform quantitative analysis would achieve little in terms of clarity. Although systematic review methodology was observed, synthesis of the evidence took the form of a narrative review. The narrative synthesis was constructed with a specific focus on the need of the aesthetic practitioner to understand the evidence for the oncologic safety of PBM in clinical practice.

## RESULTS

**Figure 1.**
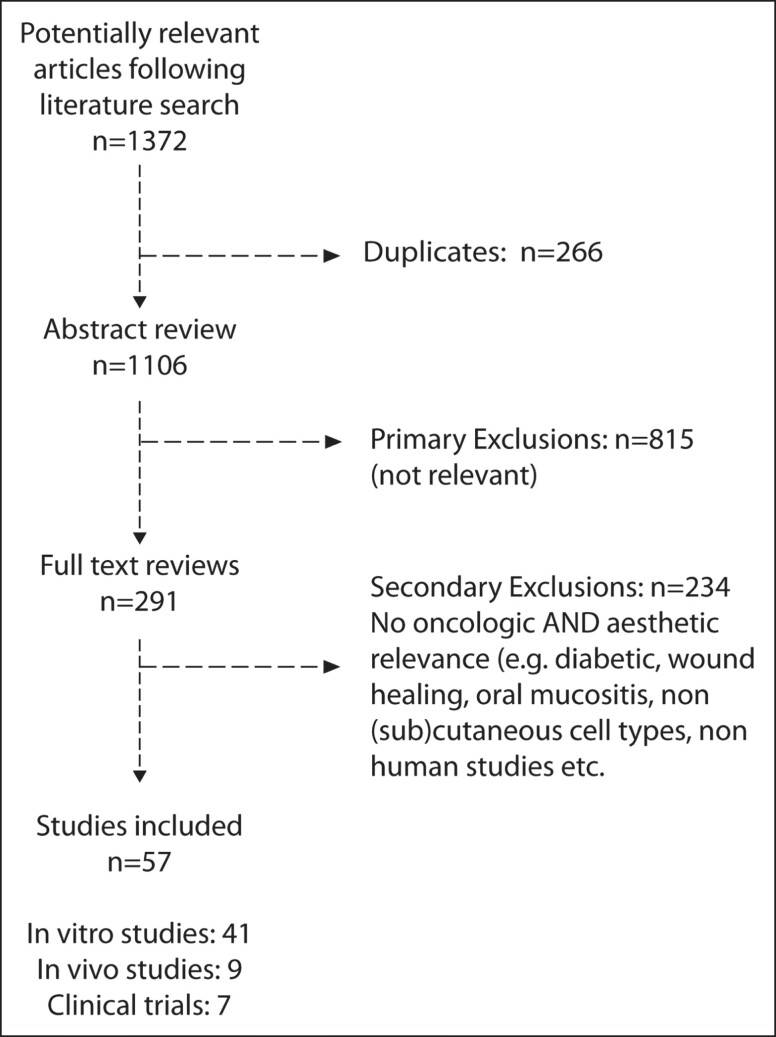
Study selection.

A total of 57 papers were included for analysis, comprising 41 in vitro studies, 9 in vivo studies, and 7 clinical trials.^20-74^ Study selection is shown in Figure 1.

**Table 1.** Summary of Evidence for Effects of PBM on Normal Human Cells In Vitro

Reference	Year	Human cells	Source	Light source	Λ (nm)	Power (mW)	Fluence (J/cm^2^)	PBM protocol	Proliferation	Migration	Viability	Notes
Chen et al^20^	2021	Fibroblasts	Human embryos	Laser	635	40	1.5, 3, 6, 12	3 exposures Cells cultured in high-glucose medium to induce oxidative stress	Increased	NA	PBM attenuated oxidative stress and inhibited apoptosis	Optimized at 3J/cm2
Flores Luna et al^21^	2020	HFF1 cells	Human dermis	Laser	660	40	0.45-3.61	1-3 exposures	Increased at 0.45 and 0.75J/cm^2^	NA	Enhanced at 0.45 and 0.75J/cm^2^	
Khorsandi et al^22^	2020	Fibroblasts	Dermis	Laser	660	?	3	1 exposure ± Gallic acid	NA	NA	No change	
Diniz et al^23^	2020	HaCat	Keratinocytes	Laser	660	?	11.7	one exposure	NA	NA	Enhanced cisplatin-induced apoptosis	
Mignon et al^24^	2018	Reticular and papillary Fibroblasts	Human dermis	LED	450, 490, 550,590, 650, 850	?		One exposure	Decreased at 450 and 490 nm; No change at ≥550nm	NA	Inhibitory at 450 nm and 30J/cm^2^; stimulatory at 850 nm and 20J/cm^2^	Reticular fibroblasts more sensitive than papillary fibroblasts
Basso et al^25^	2016	Fibroblasts	Human oral cavity	Laser	780	25	0.5-3	3 exposures	Increased	Increased	NA	Migration and proliferation attenuated by high doses of pro-inflammatory cytokines
Esmaeelinejad et al^26^	2014	Fibroblasts	Human dermis	Laser	632.8	1.5	0.5, 1, 2	3 exposures Cells cultured in standard or high glucose media	Increased at 0.5 and 1J/cm^2^ (standard) and at 0.5, 1 and 2J/cm^2^ in high glucose media	NA	No change in standard glucose; Enhanced in high glucose	
Basso et al^27^	2013	HaCat	Keratinocytes	Laser	780	40	0.5-7	3 exposures	Increased from 0.5-5J/cm^2^	NA	NA	Enhanced expression of Col1A and VEGF to 1.5J/cm^2^
Mvula et al^28^	2010	ADSC	Human adipose tissue	Laser	636	110	5	One exposure Cells cultured with EGF	Increased	NA	No change	Cell proliferation optimized with EGF
Mvula et al^29^	2008	ADSC	Human adipose tissue	Laser	635	50	5	One exposure	Increased	NA	No change	PBMT increased β1-integrin expression
Hawkins & Abrahamse^30^	2007	CRL1502 cells	Human fibroblast cell line	Laser	632.8 830 1064	?	5	One exposure	Increased	NA	NA	633 nm more effective with no background light
Hawkins & Abrahamse^31^	2006	CRL1502 cells	Human fibroblast cell line	Laser	632.8	?	0.5-16	Two exposures	Increased at 5J/cm^2^ Decreased above 10J/cm^2^	Increased at 5J/cm^2^	Increased at 5J/cm^2^ Decreased above 10J/cm^2^	
Webb & Dyson^32^	2003	Fibroblasts	Human dermis and hypertrophy scar	Laser	880	16	2.4, 4	One exposure	Decreased	NA	NA	
Kreisler et al^33^	2002	Fibroblasts	Human gingiva	Laser	809	10	1.96-7.84	1-3 exposures	Increased	NA	No change	All exposures increased proliferation initially
Almeida-Lopes et al^34^	2001	Fibroblasts	Human gingiva	Laser	670, 692, 780, 786	10-50	2	One exposure	Increased	NA	No change	Optimal increase at 780 nm in nutritionally deficient medium
Grossman et al^35^	1999	Keratinocytes	Human foreskin	Laser	780	6.5	0-3.6	One exposure	Increased at 0.45-0.95J/cm^2^	NA	No change within optimized range	Enhanced proliferation attenuated by antioxidants
Webb et al^36^	1998	Fibroblasts	Human dermis	Laser	660	17	2.4, 4	One exposure	Increased	NA	NA	Fibroblasts derived from hypertrophic scars
Pogrel et al^37^	1997	Keratinocytes	Human foreskin	Laser	830	5-100	0.12-4.24	Various exposure profiles	No change	No change	No change	No PBM identified in this study

ADSC, adipose-derived stem cells; COL1A, collagen-1-A; EGF, epidermal growth factor; LED, light-emitting diode; NA, information not available; PBM, photobiomodulation; VEGF, vascular endothelial growth factor.

### In Vitro Studies

#### The Influence of PBM on Normal Human Cell Behavior in Culture

The features of normal human cell behavior in culture that might be altered by PBM include proliferation, migration, differentiation, and viability. The present research identified 18 relevant in vitro studies in the last 25 years that utilized primary human cells or cell lines of interest in the context of skin rejuvenation; in which PBM was investigated by exposing the cells in culture to monochromatic light with or without other alterations in the culture protocols; and which examined one or more of these 4 cellular responses. The important light variables included light source, wavelength, total energy density per exposure, and number of exposures. The cells utilized were most commonly fibroblasts and keratinocytes. The culture conditions sometimes included the addition of pharmacologic or physiologic agents to the culture medium to permit examination of the influence of PBM in the presence of another variable.

Seventeen of 18 studies employed laser light, and a single study utilized LED light. Wavelengths of 450 to 880 nm were examined, although most studies used laser light at between 630 and 660 nm. In 13 of 18 studies enhanced cell proliferation under at least one PBM parameter was observed. In the 3 studies in which diminished cell proliferation in at least one PBM parameter was observed, 1 was the study employing LED light at 450 and 530 nm (wavelengths above 530 nm appeared to exert no effect on cell proliferation), 1 utilized infrared laser light (880 nm), and in the third diminished cell proliferation with a high-energy density (≥10J/cm^2^) of red light (632.8 nm) was observed. Two of the 3 studies in which migration was evaluated reported enhanced cell migration within a narrow energy density of 0.5-5J/cm^2^. Of the 13 studies in which cell viability was evaluated, 6 described changes in cell viability associated with PBM, either in isolation or in the context of a second experimental variable, whereas no change was observed in 7. A summary of the evidence for the effects of PBM on human cells in culture is presented in Table 1.

#### The Influence of PBM on Neoplastic Human Cell Behavior in Culture

In the context of PBM, relevant neoplastic cell behaviors that can be investigated in vitro include cell viability, proliferation, and invasiveness potential, which include cell migration and the expression of gene products that might serve to confer invasiveness. A total of 23 relevant studies with 34 relevant cell types were identified that met the search criteria. In these studies, 16 head and neck (including oral) squamous cell carcinoma cell lines, 10 breast adenocarcinoma or ductal carcinoma cell lines, 6 melanoma cell lines, and 2 laryngeal carcinoma cell lines were investigated. In four studies, LED light sources were employed, and the remainder used lasers. Although blue (410 nm) and infrared (1064 nm) light were investigated as part of the protocols of some studies, in most studies the effects of low-level red and near infrared light were examined, as was done in the in vitro studies on normal human cells.

In 16 studies with 25 cell lines, the influence of PBM on neoplastic cell viability was examined. PBM was found to reduce the viability of 13 of 25 neoplastic cell lines either in isolation or in the presence of another experimental agent, and PBM was found not to influence the viability of a further 8 cell lines. Of the remaining 4 cell lines, the viability of 3 was found to be dependent on total energy per unit area (fluence), whereas the viability of the remaining cell line was dependent on wavelength of light, exhibiting reduced viability when exposed to blue light only.

In 14 studies with 21 cell lines, the effect of PBM on neoplastic cell proliferation was investigated. The results were mixed. PBM suppressed proliferation in 5 experimental protocols and made no observable difference in another 5. PBM enhanced cell proliferation in 3 experimental protocols. In the remaining 8 experimental protocols, the effect of PBM was observed to vary depending on the experimental parameters, particularly total energy density (fluence) on 5 occasions. In single experimental protocols, cellular proliferation was noted to be dependent on total energy (number of light exposures), wavelength of light, and a mix of wavelength and fluence respectively.

In 4 studies with 4 cell lines, the effect of PBM on the invasiveness potential of neoplastic cells was examined; 3 by way of cell migration assays and 1 by way of investigating differential gene expression. In 2 studies it was concluded that PBM reduced neoplastic cell migration; specifically, red and near infrared light in two studies and blue light in the third. In one study red light appeared to enhance cell migration but the energy density utilized to achieve this result was unclear. In the remaining study, red light (660 nm) was associated with increased expression of MMP-9 and reduced expression of E-cadherin which, together, increase the potential for cell mobilization within the extracellular matrix. A summary of the evidence for the effects of PBM on neoplastic cells in culture is presented in Table 2.

**Table 2.** Summary of Evidence for Effects of PBM on Neoplastic Cells In Vitro

Reference	Year	Human cells	Source	Light source	Λ (nm)	Power (mW)	Fluence (J/cm^2^)	PBM protocol	Proliferation	Invasiveness potential	Viability	Notes
Austin et al^38^	2022	A375 MNT-1	Melanoma Melanoma	LED LED	633 633	? ?	640 1280 640 1280	One exposure One exposure	Decreased (1280 > 640) Decreased	NA NA	PBM enhanced apoptosis at 1280J/cm^2^ PBM enhanced apoptosis at 1280J/cm^2^	PBM enhanced ROS production in tumor cells PBM enhanced ROS production in tumor cells
Mansourian et al^39^	2022	Primary tumor sample	Oral SCC	Laser	635 810 940	200	4	One exposure	NA	NA	PBM reduced cell viability at 24 and 48 hrs (810 > 940 > 635 nm)	PBM increased IL-6 expression
Khorsandi et al^22^	2020	MDA-MB-231 A375	Breast DC Melanoma	Laser Laser	660 660	? ?	3 3	One exposure ± GA One exposure ± GA	NA NA	NA NA	PBM enhanced GA-induced cell death PBM enhanced GA-induced cell death	PBM enhanced ROS production in tumor cells PBM enhanced ROS production in tumor cells
Kianmehr et al^40^	2020 2020	SK-MEL-37 A375	Melanoma Melanoma	Laser Laser	660 660	? ?	1-6 1-6	One exposure ± *P*-coumaric acid One exposure ± *P*-coumaric acid	NA NA	NA NA	Decreased SK-MEL-37 (but not dermal fibroblasts) Decreased A375 (but not dermal fibroblasts)	Pretreatment with PBM most effective Pretreatment with PBM most effective
Diniz et al^23^	2020	SCC25 HN12	Head & neck SCC × 2	Laser	660	60	11.7	Single exposure ± Cisplatin	NA	NA	Decreased viability after 12 hrs in presence of cisplatin	PBM enhanced cisplatin-induced apoptosis
Shakibaie et al^41^	2020	MCF7	Breast AC	LED	435, 629	?	17.5 (435 nm); 7.9 (629 nm)	5 exposures	NA	NA	Decreased at 435 nm; No change at 629 nm	Did higher fluence at 435 nm influence result?
Kiro et al^42^	2019	MCF7 CSC	Breast AC MCF7 subpopulation	Laser Laser	636 825 1060 636 825 1060	75 94 75 75 94 75	5, 10, 20, 40 5, 10, 20, 40	1 exposure 1 exposure	Increased Increased	NA NA	No change No change	Increased proliferation observed at all wavelengths and fluences Increased proliferation observed at all wavelengths and fluences
Dias Schalch et al^43^	2019	SCC9	Oral SCC	Laser	660 780	40, 70	4	1 exposure	No change	Decreased migration at 660 and 780 nm	Decreased viability at 660 and 780nm	Apoptosis mechanism activated by PBM
Matsuo et al^44^	2019	HSC-3	Oral SCC	LED	630	?	?	1 exposure	No change	Increased migration	NA	Reversed by IL-6 inhibition
Bamps et al^45^	2018	SCC154, SCC61, SQD9	Head & neck SCC × 3	Laser	830	150	1-2	1 exposure	Increased at 1J/cm^2^; No change at 2J/cm^2^	NA	No change	
Ramos-Silva et al^46^	2016	MDA-MB-231	Breast DC	Laser	660	40	30 -150	1 exposure ± IR (γ radiation at 2.5, 10 Gy)	NA	NA	PBM did not enhance the viability of irradiated cells	PBM did enhance viability of irradiated fibroblast controls
Dias Schalch et al^47^	2016	SCC9	Oral SCC	Laser	660 780	10-150	2-6	1 exposure	No change	Decreased migration optimized at 70 mW and 4J/cm^2^	Decreased viability optimized at 40 mW and 4J/cm^2^	PBM induced apoptosis (optimized at 30 mW and 2J/cm^2^)
Cialdai et al^48^	2015	MCF7 MDA-MB-361	Breast AC Breast DC	Laser	808 905	?	9	3 exposures	No change	NA	No change	Fibroblast controls
Gomes-Henriques et al^49^	2014	SCC25	Oral SCC	Laser	660	?	0.5, 1	2 exposures	Increased at 1J/cm^2^	Increased: MMP9↑ E-cadherin ↓	NA	No effect at < 1J/cm2
Sperandio et al^50^	2013	SCC9 SCC25	Oral SCC × 2	Laser	660 780	40	2.05-6.15	1 exposure	NA	NA	Decreased viability of both cell types at both wavelengths by 72 hrs	PBM induced increased viability of dysplastic oral keratinocytes
Magrini et al^51^	2012	MCF7	Breast AC	Laser	633	1.3-15	0.05-1	1 exposure	NA	NA	Decreased at 0.05J/cm^2^ Increased at 1J/cm^2^	Findings attributed to dose specific changes in cell metabolism
Schartinger et al^52^	2012	SCC25	Oral SCC	Laser	660	?	?	3 exposures of 15 minutes	Decreased	NA	Decreased	Fibroblast controls exhibited increased proliferation
Powell et al^53^	2010	MCF7 MDA-MB-435S	Breast AC Melanoma	Laser Laser	780 830 904 780 830 904	50 30 90 50 30 90	0.5-12 0.5-15 0.5-15 0.5-12 0.5-15 0.5-15	1-3 exposures 1 exposure	Overall increase with 1 exposure and decreases with 3 exposures at 780 nm; overall increase up to 4J/cm^2^ at 830 and 904nm Increased at 1J/cm^2^ for 780 and 904 nm; reduced at 2-3J/cm^2^ for 830nm	NA NA	NA NA	Results are cell- and dose-specific
De Castro et al^54^	2005	KB cells	Oral SCC	Laser	635 830	31 34.5	4	2 exposures	NA	NA	Decreased	Control cells also demonstrated decreased viability
Kreisler et al^55^	2003	Primary tumor sample	Laryngeal SCC	Laser	809	10	1.96-7.84	Single exposure	Increased	NA	NA	Enhanced proliferation lasted 72 hrs post exposure
Pinheiro et al^56^	2002	HEp-2	Laryngeal SCC	Laser	635 670	5	0.04-0.48	7 exposures	Increased at 670nm	NA	NA	No change at 635nm
Sroka et al^57^	1999	ZMK MCF7	Oral SCC Breast AC	Laser	410, 488, 630, 635, 640, 830, 1064	0-20	?	One exposure	Decreased to 8J/cm^2^, then plateaued	NA	NA	Decreased mitotic rate independent of wavelength
Schaffer et al^58^	1997	ZMK	Oral SCC	Laser	805		2-20	One exposure	Decreased at 4 & 20J/cm^2^	NA	NA	No change at 2 J/cm^2^

AC: adenocarcinoma; DC: ductal carcinoma; GA: gallic acid; IR: interventional radiology; LED: light-emitting diode; MMP: matrix metalloproteinase; NA: information not available; PBM: photobiomodulation; ROS: reactive oxygen species; SCC: squamous cell carcinoma.

#### Evidence for Pro-oncogenic Effects of PBM on Normal Human Cells in Culture

Having evaluated the influence of PBM on normal and neoplastic human cells, evidence was sought to establish whether PBM has been implicated in dysplastic or neoplastic change in healthy human cell lines. No human cell in vitro experimental evidence was found to support this view.

### In Vivo Preclinical Studies

#### The Influence of PBM on (Solid) Tumor

Evidence was next sought for the effect of PBM on established tumors. By definition, it was not possible to restrict this part of the investigation to human tissue alone, and therefore all animal models were considered, as long as it was possible to conceive that the tumor under investigation might theoretically be stimulated by cutaneous light exposure. Hence, visceral tumors and non–solid-state tumors were excluded. A total of 10 relevant models were described in 9 papers. Three models utilized nude mice as the host species for human tumors, 4 were mouse tumors, 2 were hamster tumors, and 1 was a tumor in a rat. The tumor investigated was squamous cell carcinoma in 5 models (oral in 4; skin in 1), breast carcinoma in 2, melanoma in 2, and thyroid carcinoma in the remaining model. Laser-based light was employed in 8 of 10 models, with LED-based light used in the remaining 2. Again, red and/or near infrared light was utilized in all cases, whether or not other wavelengths were also part of the investigative protocol. Four models in 3 studies also introduced additional variables (radiotherapy, imiquimod, interferon-α receptor knockout mice), which were not considered in detail. The tumor characteristic studied included tumor growth in 6, tumor grade in 2, and tumor gene expression in 1. The remaining model was utilized to study host survival.

Of the 6 studies in which tumor growth was investigated, in 3 it was concluded that PBM with red and/or near infrared light did not influence tumor growth, whereas in 1 the conclusion was that PBM inhibited tumor growth (a second study reported that blue light at 405 nm inhibited tumor growth but red light did not). In 2 studies it was found that PBM increased tumor growth. In one study, immunocompromised mice were the hosts for a human tumor and in the other, the energy density associated with tumor growth was in excess of 1000J/cm^2^, which, being very much higher than in any of the other studies, was an outlier in light parameters.

Of the 2 studies in which tumor grade was examined, the conclusion in 1 was that PBM increased tumor grade, and in the other it was concluded that it made no difference. In the study in which tumor gene expression was explored, it was observed that PBM was implicated in the expression of genes associated with cell death through the synthesis of reactive oxygen species. In the study in which host species survival was considered, PBM made no difference. The evidence for the influence of PBM on solid tumor obtained with in vivo preclinical models is summarized in Table 3.

**Table 3.** Summary of Evidence for Influence of PBM on Solid Tumor In Vivo

Reference	Year	Host species	Neoplastic cell species	Cell line	Cell source	Light source	Wavelength (nm)	Power (mW)	Fluence (J/cm2)	Other variables investigated	PBM protocol	Tumor characteristic studied	Results	Notes
Barasch et al^59^	2020 2020	mouse mouse	human human	Cal-33 Cal-33	Oral SCC Oral SCC	laser LED	660 660 & 850	75 2000	18.4 3.4	Radiotherapy Radiotherapy	PBM alone vs RT alone vs PBM + RT RT ± PBM	Tumor growth Survival	No difference between RT alone and RT + PBM PBM did not change survival	PBM had no effect on tumor growth
Petrellis et al^60^	2017	rat	rat	Walker 256	Breast carcinoma	laser	660	100	35.7 107.1 214.3	NA	3 exposures (alternate days)	Gene and Inflammatory marker expression	Gene and inflammatory markers indicative of cytotoxicity at 35.7J/cm^2^	Attributed to increased ROS synthesis
Ottaviani et al^61^	2016	mouse	mouse	B16F10	Melanoma	laser	660 800 970	100 1000 2500	3 6 6	IFN-α receptor knockout mice also used	4 exposures	Tumor growth	All 3 protocols suppressed tumor growth by day 14 post exposure	PBM enhanced immune surveillance through the IFN-α receptor pathway
Rhee et al^62^	2016	mouse	human	FRO	Anaplastic thyroid carcinoma	laser	650	2	15 30	NA	One exposure	Tumor growth (and histologic features)	Tumor size, macrophage infiltration and HIF-1α, VEGF and *P*-Akt increased with irradiance.	Immune compromise model
Khori et al^63^	2016	mouse	mouse	4T1, ATCC-CRL-2539	Breast carcinoma	laser	405 532 632	1-3	unclear	NA	10 exposures over 3 weeks	Tumor growth, serum tumor markers	Tumor suppression at 405 nm; no difference at 532 & 632 nm	405 nm modulated tumor markers
De C. Montiero et al^64^	2013	hamster	hamster	Induced (DMBA)	Oral SCC	laser	660	50	95	imiquimod	14 exposures (alternate days for 4 weeks) ± imiquimod 3 times weekly	Tumor grade (histologic features)	Imiquimod improved tumor grade and inflammatory infiltration. Addition of PBM made no difference	PBM did not influence immune surveillance of oral SCC
Myakishev-Rempel et al^65^	2012	mouse	mouse	Induced (UV radiation)	Skin SCC	LED	760	unclear	2.5	NA	74 exposures (2 × daily for 37 days)	Tumor growth	PBM did not affect tumor growth	
De C. Montiero et al^66^	2011	hamster	hamster	Induced (DMBA)	Oral SCC	laser	660	30	56.4	NA	14 exposures (alternate days for 4 weeks)	Tumor grade (histologic features)	PBM caused a shift toward dedifferentiation of oral SCC	
Frigo et al^67^	2009	mouse	mouse	B16F10	Melanoma	laser	660	50	150, 1050	NA	3 exposures	Tumor growth	Increase tumor growth at 1050J/cm^2^	Very high dose used

DMBA: dimethylbenzanthracene; HIF: hypoxia-inducible factor; IFN: interferon; LED: light-emitting diode; NA: information not available; PBM: photobiomodulation; ROS: reactive oxygen species; RT: radiotherapy; SCC: squamous cell carcinoma; UV: ultraviolet; VEGF: vascular-endothelial growth factor.

#### The Influence of PBM on Normal Tissue

No evidence was found to associate PBM with neoplastic change in healthy tissue.

### Clinical Trials

In the clinical arena, PBM has been adopted extensively for the management of skin and mucous membrane irritation following radiotherapy for head and neck and breast cancers without evidence of pro-oncogenic effects.^75-78^ PBM is also utilized for skin rejuvenation, subcutaneous lipolysis, hair loss, and the management of pain and sensitivity associated with dental bleaching. To confirm safety, including oncologic safety of PBM for clinical aesthetic use, adverse event reports from clinical trials were sought.

#### Adverse Event Reporting in Clinical Trials of PBM for Aesthetic Use

The efficacy and safety of PBM for subcutaneous fat reduction, the treatment of alopecia, and pain associated with dental bleaching have been the subject of recent systematic reviews. No evidence was found to support the view that PBM was clinically harmful in general or pro-oncogenic in particular in any of these review studies.^79-81^

Seven clinical trials of PBM for skin rejuvenation were identified. Mild, transient erythema was reported in a total of 4 patients. No other adverse events were reported in any of these trials. These findings are summarized in Table 4.

**Table 4.** Summary of Adverse Event Reporting in Clinical Trials of PBM for Skin Rejuvenation

Reference	Year	Aesthetic focus	Parameter	Study design	Light source	Wavelength (nm)	Power (mW)	Fluence (J/cm2)	Other variables investigated	PBM protocol	Efficacy	Safety
Guermonprez et al^68^	2020	Skin rejuvenation	Skin surface topography and clinical examination	Prospective cohort study Split face	LED	440 660 780	NA	NA	None	24 exposures over 28 days PBM + “serum” vs “serum” only	PBM + serum improved skin topography (wrinkles, texture) over serum alone	No adverse effects
Wunsch et al^69^	2014	Skin rejuvenation	Wrinkle reduction	DB RCT	Filtered polychromatic light	Multiple (611-650)	NA	8.5-9.6	None	Two different treatment protocols vs controls	Light enhanced dermal collagen production (measured by US) and improved blinded appearance of skin	Temporary reddening of scar in one case, resolved after 1 week
Tanaka et al^70^	2011	Skin rejuvenation	Dermal elastin and surface topography	Prospective cohort study	Laser	1064	NA	14	None	PBM vs placebo	PBM enhanced dermal elastin production (histopathologic assessment) and improved skin topography (pores, texture, wrinkles)	No adverse effects
Shaoul & Mulholland ^71^	2011	Skin rejuvenation	Clinical grading of photodamage and wrinkles	Prospective cohort study	LED	“red” “near infrared”	NA	NA	None	8 biweekly exposures over 4 weeks	PBM improved photodamage over 3 months in almost all cases according to blinded photographic assessment	No adverse events
Sadick ^72^	2008	Skin rejuvenation	Clinical grading of photodamage using Glogau scale	Prospective cohort study	LED	633 830	NA	126 66	None	Alternate exposures 2 × weekly over 4 weeks	PBM improved photodamage over 12 weeks in most cases (nonblinded)	Mild facial erythema in 3 of 16 patients
Russell et al^73^	2005	Skin rejuvenation	Skin surface topography (profilometry)	Prospective cohort study	LED	633 830	NA	126 66	None	9 exposures over 29 days. Evaluations up to 12 weeks	PBM improved surface profilometry	No adverse effects
Weiss et al^74^	2005	Skin rejuvenation	Skin histopathology and surface topography	Prospective cohort study	LED	590	NA	0.1	None	8 exposures over 4 weeks. Evaluations up to 12 months	PBM enhanced dermal collagen production and improved skin topography	No adverse effects

DB: double-blind; LED: light-emitting diode; NA: information not available; PBM: photobiomodulation; RCT: randomized controlled trial; US: ultrasound.

## DISCUSSION

Photobiomodulation enhances mitochondrial ATP production and alters the reduction oxidation state of the intracellular microenvironment, features which alter the behavior of the cell.^15^ An enhanced understanding of the physiologic mechanisms underpinning PBM has given rise to the exploitation of light for clinical therapeutic and commercial benefit. However, theoretic concerns about the oncologic safety of PBM deserve to be addressed.^82^ Moreover, the myriad of variables, including light source, wavelength, fluence (energy density), and total energy (a factor of energy density and number of exposures) has contributed to a somewhat clouded picture. To comprehensively address the issue of oncologic safety requires a multifaceted review of the voluminous data being produced to help us understand PBM. The most clinically relevant means of doing so is to review the safety data from clinical trials (as has been done in the context of the clinical scenarios listed above), but in this case, to answer the question more comprehensively, the decision was made to then work backward, sequentially, to studies of the effect of PBM on solid tumors in an experimental setting and from there the effect of PBM on neoplastic, dysplastic, and finally healthy human cells.

The findings of the endeavor were as interesting as they were, in some ways, unexpected. The finding that PBM potentiates the proliferation, viability, and migratory potential of primary human cells, at least within established parameters of wavelength and energy density, was predictable, given that PBM is exploited for aesthetic skin rejuvenation, wound healing, and scar management.^83^ The variations in experimental protocols provide ample room to explain differences in observed responses to PBM. A number of light parameters appear crucially important in the efficacy of PBM, and variations in these parameters may account for variations in clinical activity. Specifically, because the chromophore is bound to cellular mitochondria, it has been observed that cells with higher numbers of mitochondria (such as skeletal muscle) tend to respond better to lower doses of light, whereas cells with lower numbers of mitochondria (such as fibroblasts and keratinocytes) require higher doses.^3^

What was more surprising were the findings that PBM either reduced or at least appeared to have no influence in most experimental protocols designed to study neoplastic cell viability, and reduced or appeared to have no influence on half of experimental protocols designed to study neoplastic cell proliferation and migration respectively. It is true that these experimental protocols were, in some cases, different in that PBM was investigated in the presence of a second variable in 6 of the 24 experimental protocols. Nevertheless, PBM was implicated in reactive oxygen species–induced cell damage and apoptosis in several different experimental protocols. Additionally, no experimental evidence was found to suggest that PBM can induce neoplastic change in healthy cells. Although it is easy to overlook this negative finding, this is nonetheless important. Following exposure to LLLT, healthy cells stay healthy. The equivocal findings of this part of the study echo those of a recent review that looked specifically at the tumor modulating effects of PBM on head and neck squamous cell carcinoma. The author of this study concluded that the evidence failed to support a clear conclusion.^84^

Frankly, it is important not to read too much into in vitro culture models.^85^ Crucially, they do not adequately represent the relatively greater complexity of the in vivo tissue microenvironment, a dynamic system characterized by complex interactions between different cells based on molecular cross signals coordinated by cells of innate and adaptive immunity. A total of 10 experimental protocols in 9 papers examined the influence of PBM on solid tumors in a range of animal models. The results require careful consideration. In brief, 4 of 6 models that examined tumor growth concluded that PBM either suppressed tumor growth or, more commonly, made no difference. The 2 experimental protocols that observed an increase in tumor growth in response to PBM might reasonably be considered nonrepresentative. In one case, the energy density was over 1000J/cm^2^, which is very much higher than the energy density typically defined for low-level light therapy. The other, by employing nude mice to host human tumors, eliminated the crucial role of host immune surveillance in the biological response to tumor. This was 1 of 3 experimental protocols that employed nude mice. Of the other 2, in 1 study no difference was observed in tumor survivability using PBM, and in the other PBM did not attenuate radiotherapy-induced tumor shrinkage. Among immune-normal in vivo models, the evidence synthesized in this study supports the conclusion that, with established parameters, PBM probably does not enhance tumor growth or virulence, nor does it induce cancer in normal tissue.

Previously published reviews of clinical trials have concluded that PBM may be useful in attenuating some of the collateral damage of oncologic therapy without diminishing the efficacy of the oncologic therapy.^76-78^ In fact, it may even play a supportive antitumor role.^7^ It would appear that it does so by priming the cellular response to oxidative stress, helping to mitigate the destructive effects of reactive oxygen species induced by radiation or chemotherapy.^86,87^ That said, it would be wrong to assume that the role of PBM in oncologic management is limited solely to the management of the side effect profiles of standard oncologic therapy. Near infrared light is currently attracting interest for utilization with light-activated nanoparticle dispersal systems for precision therapeutic delivery in the management of melanoma.^88,89^ The abundance of evidence for the utilization of PBM as a means of managing the side effects of chemoradiotherapy, including oral mucositis, radiation dermatitis, and lymphedema, is a separate issue, and it would be unwise to draw conclusions about oncologic safety based on the findings that PBM can be effective in the management of the side effects of cancer therapy. Nevertheless, a recent systematic review of all of the clinical and preclinical evidence reached a similar conclusion to the present focused study.^90^ Moreover, recent systematic reviews on the use of PBM for the enhancement of osseointegration of dental implants and pain associated with dental analgesia yielded mixed results in terms of efficacy but, again, no evidence of harmful side effects.^91,92^ However, as in the case of aesthetic practice, an accurate appraisal of the role of PBM in clinical dentistry is noted to be hampered by inconsistencies in the PBM parameters utilized.^93,94^

The alternative way to review the findings of this study is that the quality of clinical evidence is simply not good enough to be able to accurately define the risks and limitations of PBM. Although this might be true, PBM has now become so widespread that it might reasonably be anticipated that a harmful incident would have come to light if PBM had the potential to cause harm. Additionally, it is worth mentioning again that the earliest studies of PBM were looking for evidence of harm and did not find any. What this study adds to the existing knowledge base is confirmation of the lack of adverse events reported in clinical trials and case series of PBM for aesthetic use, and a comprehensive summary of in vitro data from human cells that largely supports the conclusion that primary and neoplastic cells behave differently when exposed to low-level light radiation.

There are a number of limitations to this study. Most of the clinical evidence of efficacy and safety in aesthetic clinical practice comes from methodologically flawed, single-center clinical trials and case series. Well-designed, large scale multicenter randomized controlled trials (RCT) of efficacy and safety would be very welcome, but the likelihood is remote at present. There may be cause for optimism that, with the accumulation of evidence from small clinical trials, sufficient momentum may be gained to support funding for an RCT. The number of parameters involved continues to make interpretation of the results challenging. It is for this reason that this review and others have adhered to a narrative review structure, without employing the tools that define high quality systematic reviews of clinical trials such as a risk of bias, heterogeneity assessments, and sensitivity analyses. For the same reason, this review does not employ any statistical analytic tools, because there are too many experimental variables to accommodate within quantitative analytic models for any conclusions drawn to be statistically meaningful.

## CONCLUSIONS

PBM is increasingly being utilized at home to supplement office-based surgical and nonsurgical aesthetics. It is important that we are in a position to counsel our patients wisely, and to do so in the context of PBM in a patient who has previously had cancer places a special demand on us to understand the evidence. Without a means of navigating the disparate strands of evidence, clinicians may feel uncomfortable about providing an opinion. Despite theoretical concerns there is no clinical evidence to support the proposition that PBM should be avoided in patients who are being treated for cancer, previously had cancer, or have risk factors for cancer. Care must be taken not to read too much into in vitro and in vivo studies. Nevertheless, an evaluation of these data suggests that the behavior of neoplastic cells and solid tumor in response to PBM differs from the behavior of healthy cells and is much more likely to be, although not always, inhibitory. Although clinical advice is always based on evidence that may be subject to revision over time as the body of evidence evolves, the reasonable conclusion from the evidence we currently have at our disposal is that PBM is safe for aesthetic purposes, including for patients with an oncologic history.

## Supplementary Material

sjad018_Supplementary_Data
