# Supplementary material for: Photobiomodulation: A Systematic Review of the Oncologic Safety of Low-Level Light Therapy for Aesthetic Skin Rejuvenation
Source: Aesthet Surg J. 2023 Feb 1;43(5):NP357–71. doi: 10.1093/asj/sjad018 (PMC10309024; doi:10.1093/asj/sjad018)
Supplement: sjad018_Supplementary_Data [file sjad018_supplementary_data.zip › 22-0977_Supplemental Table 1.docx]

**Supplemental Table 1:** OVID (Wolters Kluwer, Alphen van den Rijn, the Netherlands) Literature Search Strategy

| Number | Search tern |
| --- | --- |
| 1 | "Photobiomodulation" |
| 2 | “PBM” |
| 3 | “photostimulation” |
| 4 | "Low level light therapy" MeSH |
| 5 | "low level laser therapy” |
| 6 | "Light emitting diode" |
| 7 | " LED" |
| 8 | "laser" |
| 9 | “laser biostimulation” |
| 10 | 1 OR 2 OR 3 OR 4 OR 5 OR 6 OR 7 OR 8 OR 9 |
| 11 | “oncology” |
| 12 | “cancer” |
| 13 | “dysplas$” |
| 14 | “neoplas$” |
| 15 | “malignan$” |
| 16 | “tumor” |
| 17 | “tumour” |
| 18 | “proliferation” |
| 19 | “migration” |
| 20 | “viable” |
| 21 | “viability” |
| 22 | 11 OR 12 OR 13 OR 14 OR 15 OR 16 OR 17 OR 18 OR 19 OR 20 OR 21 |
| 23 | “in vitro” |
| 24 | “in vivo” |
| 25 | 23 OR 24 |
| 26 | 10 AND 22 AND 25 |
| 27 | Limit 26 to yr=”1997-current” |
| 28 | “cicatrix rejuvenation” (MeSH) |
| 29 | “rejuven*” |
| 30 | “skin” |
| 31 | “skin rejuvenation” |
| 32 | “clinical trial” |
| 33 | 28 OR 29 OR 30 OR 31 AND 32 |
| 34 | 10 AND 33 |
| 35 | limit 34 to yr="1997 – Current" |
